# Propylnitrosourea-induced T-lymphomas in LEXF RI strains of rats: genetic analysis

**DOI:** 10.1038/sj.bjc.6690432

**Published:** 1999-05

**Authors:** L-M Lu, H Shisa, J-i Tanuma, H Hiai

**Affiliations:** Department of Pathology and Biology of Disease, Graduate School of Medicine, Kyoto University, Sakyo-ku, Kyoto, 606-5801, Japan; Department of Pathology, Ehime University School of Medicine, Ehime, 791-0295, Japan; Laboratory of Pathology, Saitama Cancer Center Research Institute, Ina, Saitama, 362-0806, Japan

**Keywords:** genetic susceptibility, propylnitrosourea, carcinogen, T-lymphoma, recombinant inbred strain, rat, LEXF, QTL analysis

## Abstract

Oral administration of propylnitrosourea (PNU) in drinking water induces high incidence of lympho-haemopoietic malignancies in rats. Previously we reported that F344 strain rats were highly susceptible to T-lymphomas, and LE/Stm rats, to erythro- or myeloid leukaemias. For analysis of the genetic factors determining types of diseases, we have established LEXF recombinant inbred strains of rats comprising 23 substrains, each derived from intercross between F344 and LE/Stm rats. Rats of 23 LEXF substrains were given PNU, and the development of tumours was observed. The overall incidence of haemopoietic tumours ranged from 100% to 66.7%, and the fractions of T-lymphomas, from 100% to 4%, showing a continuous spectrum. Based on the genetic profile published as a strain distribution pattern table for the LEXF, we screened the potential quantitative trait loci involved in determination of the types of disease and length of the latency period. Statistical calculation was performed using the Map Manager QT software developed by Manly. Four loci, on chromosome 4, 7, 10 and 18, were suggested to associate with the T-lymphoma susceptibility and three loci, on chromosome 1, 5 and 16, with the length of the latency period. These putative loci were further examined in backcross (F344 × LE)F1 × LE. Among seven loci suggested by the recombinant inbred study, three loci, on chromosome 5, 7 and 10, were significantly associated with T-lymphomas and another locus on chromosome 1, just weakly. These observations indicate that PNU-induced lymphomagenesis is a multifactorial genetic process involving a number of loci linked with susceptibility and resistance. © 1999 Cancer Research Campaign


*British Journal of Cancer* (1999) **80**(5/6), 855–861
© 1999 Cancer Research Campaign
Article no. bjoc.1998.0432

### Propylnitrosourea-induced T-lymphomas in LEXF RI strains of rats: genetic analysis

**L-M Lu[1,2], H Shisa[3], J-i Tanuma[3] and H Hiai[1]**

[1]Department of Pathology and Biology of Disease, Graduate School of Medicine, Kyoto University, Yoshida-Konoe-cho, Sakyo-ku, Kyoto 606-5801, Japan; [2]Department of Pathology, Ehime University School of Medicine, Shigenobu-cho, Ehime, 791-0295, Japan; [3]Laboratory of Pathology, Saitama Cancer Center Research Institute, Ina, Saitama 362-0806, Japan

**Summary** Oral administration of propylnitrosourea (PNU) in drinking water induces high incidence of lympho-haemopoietic malignancies in rats. Previously we reported that F344 strain rats were highly susceptible to T-lymphomas, and LE/Stm rats, to erythro- or myeloid leukaemias. For analysis of the genetic factors determining types of diseases, we have established LEXF recombinant inbred strains of rats comprising 23 substrains, each derived from intercross between F344 and LE/Stm rats. Rats of 23 LEXF substrains were given PNU, and the development of tumours was observed. The overall incidence of haemopoietic tumours ranged from 100% to 66.7%, and the fractions of T-lymphomas, from 100% to 4%, showing a continuous spectrum. Based on the genetic profile published as a strain distribution pattern table for the LEXF, we screened the potential quantitative trait loci involved in determination of the types of disease and length of the latency period. Statistical calculation was performed using the Map Manager QT software developed by Manly. Four loci, on chromosome 4, 7, 10 and 18, were suggested to associate with the T-lymphoma susceptibility and three loci, on chromosome 1, 5 and 16, with the length of the latency period. These putative loci were further examined in backcross (F344 × LE)F1 × LE. Among seven loci suggested by the recombinant inbred study, three loci, on chromosome 5, 7 and 10, were significantly associated with T-lymphomas and another locus on chromosome 1, just weakly. These observations indicate that PNU-induced lymphomagenesis is a multifactorial genetic process involving a number of loci linked with susceptibility and resistance.

**Keywords:** genetic susceptibility; propylnitrosourea; carcinogen; T-lymphoma; recombinant inbred strain; rat; LEXF; QTL analysis

Our recent studies have shown that types of lymphomas and leukaemias are determined by host genes in certain experimental models (Yamada et al, 1994*a*, 1994*b*). Propylnitrosourea (PNU)-induced T-lymphomas are one such example. F344 rats exhibit high susceptibility, whereas many other strains of rats develop predominantly erythro- or myeloid leukaemias (Shisa and Hiai, 1985; Shisa and Suzuki, 1991) We have assumed that a single dominant gene of F344 rats determines the susceptibility to T-lymphomas and that another independently segregating dominant gene determines the length of the latency period (Shisa and Hiai, 1985). Subsequent efforts to map such genes with crosses between F344 and LE rats, however, have met with great difficulty (Shisa et al, unpublished observation). The difficulty seems to arise partly from paucity of rat genetic marker at that time and partly because these traits are quantitative in nature and mutigenic, rather than a single gene inheritance.

The recombinant inbred (RI) strains represent a set of stable inbred strains derived from F2 intercross between two parental inbred stains. The RI strains are extremely useful for the genetic analysis of complex traits (Taylor, 1978; Bailey, 1981). To analyse T-lymphoma susceptibility, we have established a new set of rat RI strains, LEXF, from matings between F344 and LE/Stm strains rats. The LEXF RI strains comprise 11 independent strains and 13 of their sublines. The strain distribution pattern (SDP) of LEXF has been extensively analysed (Shisa et al, 1997; Lu et al, 1998).

*Received 24 June 1998*
*Revised 21 December 1998*
*Accepted 22 December 1998*

*Correspondence to:* H Hiai

In this paper, we report the PNU-induced carcinogenesis in the LEXF RI strains, and survey the host genes responsible for determination of the disease types and length of the latency period calculated using quantitative trait locus (QTL) analysis with the Map Manager QT software developed by Manly (1993). We found that numerous QTL with opposed functions are involved in determining the types of lymphomas and latency periods. To control type I and type II errors (Belknap et al, 1992, 1996), we further evaluated these putative loci in 137 backcross generation of (F344 × LE)F1 × LE rats. Significant linkage with T-lymphomagenesis was confirmed for three loci on chromosome 5, 7 and 10 and suggestive linkage, for a locus on chromosome 1.

### MATERIALS AND METHODS

### Animals

The LE/Stm rats were originally derived from a closed colony of Long-Evans rats at Ben May Laboratory, University of Chicago (Chicago, IL, USA) and maintained at Saitama Cancer Center Research Institute by brother–sister matings for > 50 generations. The F344 rats were derived from a pair of F344/DuCrj rats purchased from Charles River Japan, Inc. (Kanagawa, Japan) and thereafter maintained by brother–sister matings for > 23 generations. The LEXF was a set of 11 independent RI strains and 13 sublines (Shisa et al, 1997) In this study all LEXF RI strains except for LEXF 6B, a poor breeder, were used. These sublines were branched out at the 7th to 11th generations after an attempt to fix coat colour. The main lines are designated either by the strain number alone or by the number plus A when any sublines exist. The sublines are denoted by the letters B–D following the strain number. Between F344 and LE/Stm, approximately 38% of genetic loci are polymorphic, and we have established the SDP of 153 loci (Shisa et al, 1997; Lu et al, 1998). Among independent substrains, the polymorphism ratio was 41~58%, and among sublines 12~18%. The backcross rats were generated by mating (F344 × LE)F1 female to LE male rats. All the rats were maintained under specific pathogen-free conditions in our animal facility, kept at 24 ± 1°C.

### PNU administration

PNU (Iwaki Kagaku Co. Ltd., Tokyo, Japan) was dissolved in deionized water at a concentration of 400 µg $ml^{-1}$ immediately before use. All the rats were allowed access to PNU water ad libitum from 17:00 h to 09:00 h, but no water was given after 09:00 h. The volume of PNU water consumed was measured daily for estimation of the amount of PNU taken up. The rats were inspected twice each week and weighed each week. The administration of PNU was started at 40 days of age and continued for 90 days. Thereafter rats were given PNU-free water. All of the rats were killed when they were moribund or at the age of 12 months, and full autopsy including histopathological examination was carried out. T-lymphomas were diagnosed by both involvement of the thymus and expression of Thy-1.1 antigen on the surface of tumour cells. Fluorescein isothiocyanate (FITC)-labelled anti-Thy1.1 antibody was purchased from PharMingen (San Diego, CA, USA). Staining and analysis of cells in a FACScan (Becton-Dickinson, Mountain View, CA, USA) were carried out as described previously (Lu et al, 1997). Other types of leukaemias, without thymus enlargement and Thy1.1 expression, were erythroid or myeloid leukaemias. Erythroid leukaemias, consisting > 95% of Thy-1.1 negative non-T leukaemias, were diagnosed by histology and cytology in stamp preparation as well as negative peroxidase staining. In contrast, myeloid leukaemia cells had azurophilic and peroxidase positive cytoplasmic granules and variable expression of Gr-1 (PharMingen).

### Genetic analysis

QTL analysis by interval mapping procedure was performed for RI strains with Map Manager QT, a computer software package developed and provided by Manly (1993). The Map Manager QT applies one-way analysis of variance designed to identify Mendelian loci that are significantly associated with quantitative trait phenotype among mapped marker loci. The likelihood of ratio statistic (LRS) calculated with the Map Manager QT is approximately equivalent to the $\chi^2$ statistic. As a means of establishing more reliable critical values for the significance of LRS generated by the interval mapping procedures, 1000 times permutation test (Manly, 1996) is built into the Map Manager QT. The minimum values of LRS for suggestive, significant and highly significant linkage are 7.8, 13.1 and 25.5 respectively. These values correspond to the suggestions made by Lander and Kruglyak (1995). Genotypes of all LEXF RI strains were based on the SDP table published previously (Shisa et al, 1997; Lu et al, 1998).

A genome wide screening was carried out for 20 backcross rats with T-lymphomas and another 20 rats with other leukaemias. Assuming a radius of the swept circle 20 cM, the coverage was 88%.

**Table 1** PNU-induced leukaemias in LEXF RI strains

| RI strains | No. of rats | All leukaemia (%) | T-lymphoma | | Other leukaemia[a] | |
|---|---|---|---|---|---|---|
| | | | (%) | Latency (Days±s.d.) | (%) | Latency (Days±s.d.) |
| F344 | 29 | 28 (96.6) | 28 (96.6) | 106.6±15.6 | 0 | |
| LE | 26 | 25 (96.2) | 3 (11.5) | 158.0±9.3 | 22 (84.6) | 144.4±19.9 |
| F1 | 25 | 25 (100) | 24 (96.0) | 126.5±25.1 | 1 (4.0) | 152 |
| 1A | 26 | 26 (100) | 24 (92.3) | 101.8±9.6 | 2 (7.7) | 121.0±1.0 |
| 1B | 26 | 26 (100) | 26 (100) | 110.5±17.7 | 0 | |
| 1C | 23 | 23 (100) | 22 (95.6) | 111.0±16.6 | 1 (4.3) | 97 |
| 2A | 21 | 21 (100) | 6 (28.6) | 107.5±8.0 | 15 (71.4) | 112.6±7.1 |
| 2B | 20 | 19 (95.0) | 18 (90.0) | 126.0±23.9 | 1 (5.0) | 103 |
| 2C | 20 | 19 (95.0) | 17 (85.0) | 128.4±25.6 | 2 (10.0) | 132.5±8.5 |
| 2D | 19 | 17 (89.5) | 2 (10.5) | 124.0±16.0 | 15 (78.9) | 126±21.3 |
| 3 | 26 | 23 (88.5) | 16 (61.5) | 130.6±8.0 | 7 (26.9) | 167.5±70.0 |
| 4 | 21 | 20 (95.2) | 12 (57.1) | 128.7±24.3 | 8 (38.1) | 150.6±17.7 |
| 5 | 19 | 17 (89.5) | 3 (15.8) | 168.0±55.7 | 14 (73.7) | 167.6±33.6 |
| 6A | 26 | 25 (96.2) | 18 (69.2) | 139.0±29.2 | 7 (26.9) | 174.1±54.1 |
| 7A | 25 | 25 (100) | 22 (88.0) | 123.7±31.7 | 3 (12.0) | 123.0±30.5 |
| 7B | 20 | 19 (95.0) | 11 (55.0) | 125.3±17.0 | 8 (40.0) | 176.1±33.1 |
| 7C | 16 | 15 (93.8) | 15 (93.8) | 136.5±23.9 | 0 | |
| 8A | 17 | 14 (82.4) | 2 (11.8) | 153.5±15.5 | 12 (70.6) | 173.2±35.2 |
| 8B | 25 | 19 (76.0) | 1 (4.0) | 114 | 18 (72.0) | 192.5±39.4 |
| 8C | 20 | 17 (85.0) | 2 (10.0) | 233.0±10.0 | 15 (75.0) | 148.6±31.2 |
| 8D | 14 | 11 (78.6) | 6 (42.9) | 139.6±13.5 | 5 (35.7) | 173.4±42.0 |
| 9 | 27 | 24 (88.8) | 3 (11.1) | 118.0±10.2 | 21 (77.8) | 144.5±32.0 |
| 10A | 30 | 28 (93.3) | 24 (80.0) | 130.7±30.5 | 4 (13.3) | 163.7±10.2 |
| 10B | 21 | 14 (66.7) | 14 (66.7) | 143.3±45.0 | 0 | |
| 10C | 21 | 21 (100) | 16 (76.2) | 141.5±32.7 | 5 (23.8) | 165.2±33.2 |
| 11 | 25 | 24 (96.0) | 24 (96.0) | 116.7±33.0 | 0 | |

[a]See description in text.

 © Cancer Research Campaign 1999

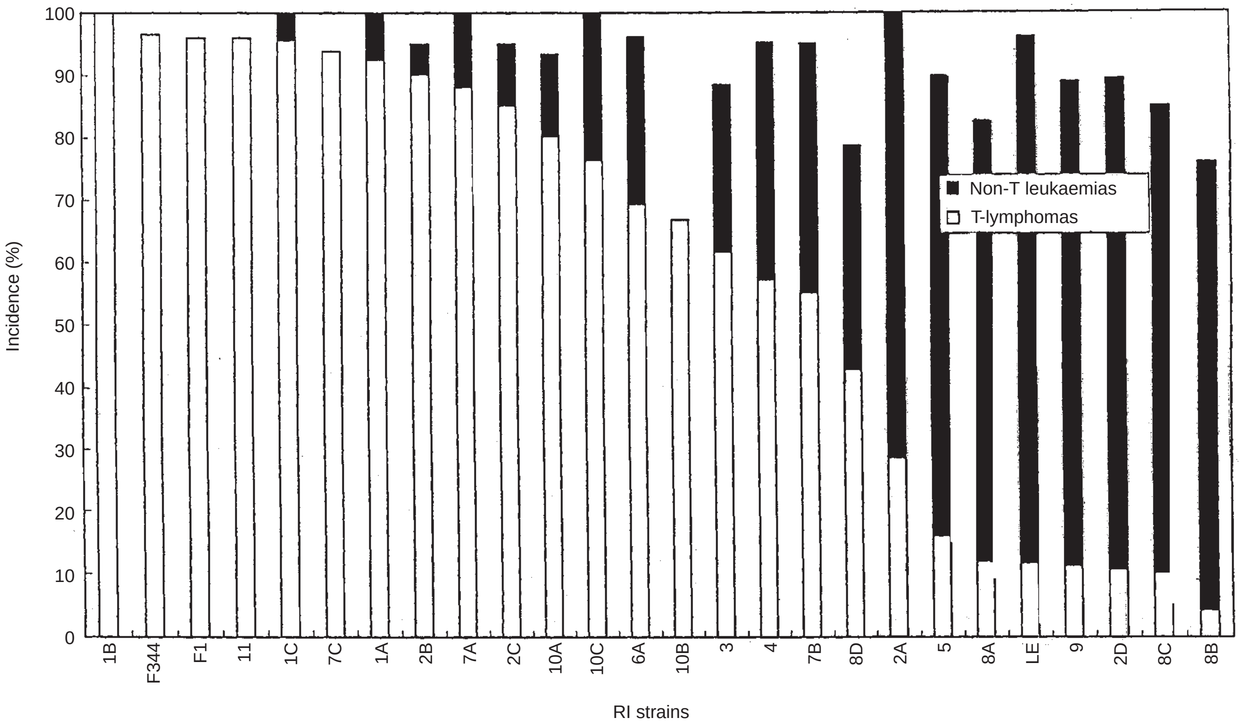

**Figure 1** Percent incidence of T lymphomas (open column) and other types of leukaemias (closed columns) in the rats of LEXF RI strains, F344, LE/Stm and (F344 × LE/Stm)F1

### Statistical analysis

Chi-square test and unpaired Student's *t*-test were used.

### RESULTS

### PNU-induced carcinogenesis in LEXF RI strains

Oral administration of PNU induced haemopoietic malignancies at a high incidence in all of the LEXF RI strain rats (Table 1). The overall incidence of leukaemias ranged from 66.7% to 100% depending on the strain. Out of 508 LEXF RI rats, 466 (91.7%) died of leukaemia. The types of leukaemias were T-lymphomas, myeloid or erythroleukaemias, and their relative frequency varied widely from one strain to another. Over 95% of non-T leukaemias were erythroleukaemias. The T-lymphomas had the shorter latency period: the latency period of 303 T-lymphomas was 132.7 ± 26.7 days, and that of 163 other leukaemias 148.0 ± 27.9 days ($P = 1.1 \times 10^{-8}$). F344 and (F344 × LE)F1 rats were highly susceptible to T-lymphomas, and LE/Stm rats to non-T leukaemias. Figure 1 illustrates the frequency of such tumours among LEXF RI strains in descending order of T-lymphoma incidence. Some LEXF RI strains, showed higher susceptibility than the parental F344, whereas that in other strains was lower than that in the LE/Stm strain. This relation as well as the continuous spectrum of

**Table 2** QTLs affecting T-lymphoma incidence

| Loci | Relative distance (cM) | LRS | Genotype of 23 RI strains % T-lymphoma incidence ± s.d. (No. of strains) | | *P* | Genotype of 11 independent RI strains % T-lymphoma incidence ± s.d. (No. of strains) | | *P* |
|---|---|---|---|---|---|---|---|---|
| | | | F | L | | F | L | |
| *D4Mgh8*[a] | | 11.6 | 36.3±33.4 (10) | 73.2±23.6 (13) | 0.005 | 31.4±29.0 (5) | 82.3±16.4 (6) | 0.004 |
| *D7Wox24* | 7.5 | 3.1 | 66.3±24.5 (13) | 42.2±40.0 (10) | 0.09 | | | |
| *D7Mit4* | | 13.1 | 77.2±14.1 (10) | 39.1±35.3 (13) | 0.004 | 84.6±13.9 (5) | 44.8±31.9 (6) | 0.03 |
| *D7Mit5* | 2 | 7.2 | 71.2±24.4 (11) | 41.1±35.9 (12) | 0.03 | | | |
| *D10Mgh3* | 13.2 | 6.2 | 44.2±34.9 (14) | 78.0±16.9 (9) | 0.01 | | | |
| *D10Mgh6* | | 6.7 | 43.5±32.7 (14) | 79.1±17.6 (9) | 0.007 | 43.4±32.1 (7) | 86.8±16.3 (4) | 0.04 |
| *D10Mgh8* | 2.3 | 6.4 | 43.8±34.6 (14) | 78.4±17.1 (9) | 0.01 | | | |
| *D18Mit5*[a] | | 11.5 | 36.3±36.1 (11) | 73.2±19.9 (12) | 0.005 | 44.0±39.5 (5) | 71.8±26.2 (6) | >0.05 |

[a]The single locus within the linkage group.

**Table 3** T-lymphoma incidence among LEXF-2 and LEXF-8 sublines and alleles of some chromosomal loci

| LEXF subline | T-lymphoma (%)[a] | Genotype at marker loci[b] | | | | | | | |
|---|---|---|---|---|---|---|---|---|---|
| | | *D4Mgh8* | *D5Mit7* | *D7Mit4* | *D10Mgh6* | *D12Mit1* | *D14Mit6* | *D16Mgh1* | *D18Mit5* |
| 2A | 28.6 | L | L | L | F | L | L | L | L |
| 2B | 90 | L | L | F | F | F | F | F | L |
| 2C | 85 | L | L | L | F | F | F | F | L |
| 2D | 10.5 | L | L | L | F | L | L | L | F |
| 8A | 11.8 | L | L | L | F | L | F | F | F |
| 8B | 4 | L | L | L | F | L | F | F | F |
| 8C | 10 | L | L | L | F | L | F | F | F |
| 8D | 42.9 | L | L | L | F | L | L | L | F |

[a]Percent incidence of T-lymphomas from Table 1. [b]Genotypes of all sublines from SDP table in Lu et al (1998). *D4Mgh8*, *D7Mit7* and *D10Mgh6* are selected from QTL analysis of lymphoma incidence in 11 independent LEXF RI strains. The other four loci are selected by apparent association of lymphoma incidence and genotype in SDP table.

**Table 4** Loci affecting the length of T-lymphoma latency

| Loci[a] | Relative distance (cM) | LRS | Genotype of RI strains[b] and T-lymphoma latency in days ± s.d. (No. of strains) | | *P* |
|---|---|---|---|---|---|
| | | | F | L | |
| *D1Mit13* | | 10.2 | 137.5 ± 5.5 (6) | 122.0 ± 10.5 (10) | 0.005 |
| *D1Wox9*[c] | 2.4 | 10.3 | 136.2 ± 5.6 (7) | 121.2 ± 10.9 (9) | 0.005 |
| *D5Mit7*[d] | 1.1 | 15.9 | 106.4 ± 6.51 (3) | 131.54 ± 7.9 (13) | 0.0002 |
| *D5Wox11* | | 10.8 | 113.7 ± 13.5 (4) | 131.8 ± 8.2 (12) | 0.006 |
| *D5Wox4* | 2.4 | 9.4 | 116.8 ± 11.3 (5) | 133.2 ± 7.0 (11) | 0.01 |
| *D16Wox9*[e] | | 14.4 | 114.5 ± 11.3 (5) | 133.2 ± 7.0 (11) | 0.001 |

[a]Marker loci within the linkage group containing the loci with highest LRS. [b]LEXF-2A, 2D, 5, 8A, 8B, 8C, 9 are excluded from calculation. [c]*D1Wox9* shared the same SDP for all 23 RI strains with *D1Wox23* and *D1Mit5*. [d]*D5Mit7* shared the same SDP pattern with *D5Mit4* localized 3.8 cM proximal. [e]*D16Wox9* shared the same SDP with *D16Wox10* and *D16Wox11*.

T-lymphoma incidence suggested that the types of tumours were determined by multiple genes. The proportions of T-lymphomas and other leukaemias generally showed a reciprocal relationship, but in the LEXF 1B, 11, 7C and 10B strains, no leukaemias other than T-lymphomas were found.

In addition to lymphoma/laeukemia, PNU induced a variety of non-haemopoietic tumours as diagnosed by histopathology. Duodenal adenocarcinoma was found in 273/508 (53.7%) LEXF RI rats. As duodenal cancers developed slightly later than lymphoma/leukaemia, most leukaemia-free rats ultimately died of duodenal cancers. Although the exact incidence was not clear because of frequent concomitant development of leukaemia/lymphoma, no apparent susceptibility difference was found among strains. Low incidence (< 5%) of liver tumours (haemangiomas and liver cell carcinomas), granulosa cell tumours of ovary, renal cell carcinomas and ear duct cancers were found in a few rats. The LEXF-9 strain was unique because mammary carcinomas developed in ten of 17 female rats. No spontaneous tumour was observed in any LEXF rats before 12 months of age.

### QTL determining T-lymphoma development

Using T-lymphoma incidence as a quantitative parameter, we surveyed potential host loci linked with susceptibility or resistance. Table 2 lists the four linkage groups containing loci with > 6.5 LRS calculated for 23 RI strains by Map Manager QT. On the left half of the Table, the average incidence of T-lymphoma in all 23 substrains is shown by either F344 or LE allele, and on the right half, that in 11 independent substrains. Out of these four loci, *D18Mit5* (chromosome 18) was excluded since the differences in T-lymphoma incidence in independent RI strains were not significant. However, the differences were significant at *D4Mgh8* (chromosome 4), *D7Mit4* (chromosome 7) and *D10Mgh6* (chromosome 10) both for all RI strains and for independent strains. At *D7Mit4*, F344-derived alleles favoured high T-lymphoma incidence ($P$ = 0.004), whereas at *D4Mgh8* and *D10Mgh6*, LE-derived alleles did.

Several LEXF strains showed considerable differences in T-lymphoma incidence among their sublines. These sublines are potentially informative in genetic analysis, because they are less different from each other than from other independent RI strains. As seen in Table 3, the T-lymphoma incidence was high in LEXF 2B (94.7%) and 2C (89.5%) but low in 2A (28.5%) and 2D (11.8%). Among LEXF 8 sublines, T-lymphoma incidence was 42.9% in 8D but far lower in 8A, 8B and 8C. Such differences were not explained by the three loci discussed above and D5Mit7, as they were not so polymorphic among these sublines. This observation suggested the existence of other critical loci segregating among these substrains. Examining the SDP table published previously (Lu et al, 1998), we found other loci, *D12Mit1*, *D14Mit6* and *D16Mgh1*, the alleles of which showed a strain distribution appar-

 © Cancer Research Campaign 1999

**Table 5** Genetic analysis of the backcross rats

| Loci | Relative distance (cM) | Genotype of backcross rats with T-lymphoma F/L | T-lymphoma L/L | Other leukaemias F/L | Other leukaemias L/L | $\chi^2$ | *P* |
|---|---|---|---|---|---|---|---|
| *c* | 17.3 | 57 | 37 | 20 | 23 | 2.4 | 0.1 |
| *D1Mit13* | 2.4 | 51 | 42 | 13 | 30 | 7.1 | 0.008 |
| *D1Mit5* | 10 | 50 | 44 | 13 | 30 | 6.3 | 0.01 |
| *D1Mgh12* | | 49 | 45 | 16 | 27 | 2.6 | 0.1 |
| *D4Mgh8* | | 50 | 44 | 16 | 27 | 3 | 0.08 |
| *D5Mit4* | 3.8 | 59 | 35 | 15 | 28 | 9.2 | 0.002 |
| *D5Mit7* | 3.5 | 64 | 30 | 10 | 33 | 23.6 | 0.000001 |
| *D5Wox4* | 2.4 | 64 | 30 | 16 | 27 | 11.6 | 0.0007 |
| *D5Mgh8* | | 60 | 34 | 18 | 25 | 5.8 | 0.02 |
| *D7Wox24* | 7.5 | 50 | 44 | 16 | 27 | 3 | 0.08 |
| *D7Mit4* | 2.0 | 56 | 38 | 9 | 34 | 17.7 | 0.00003 |
| *D7Mit5* | | 55 | 39 | 13 | 30 | 9.4 | 0.002 |
| *D10Mgh3* | 7.6 | 41 | 49 | 27 | 15 | 4 | 0.05 |
| *D10Wox6* | 5.6 | 39 | 55 | 28 | 15 | 6.6 | 0.01 |
| *D10Mgh8* | 2.3 | 37 | 57 | 32 | 11 | 14.5 | 0.0001 |
| *D10Mgh8* | | 40 | 54 | 29 | 14 | 7.3 | 0.007 |
| *D18Mit5* | | 36 | 58 | 21 | 15 | 4.2 | 0.04 |

ently associating with T-lymphoma incidence (Table 3). However, LRS for T-lymphoma incidence in both 23 substrains and 11 independent LEXF RI strains was < 6.5 at these loci (calculation not shown). To address such differences, analysis of PNU-induced tumours in crosses among these sublines is required.

### Loci determining the latent period

Subsequently we searched for genes determining the length of the latency period using the period (days) until T-lymphoma development as a quantitative parameter. In this calculation, sublines with < 3 rats bearing T-lymphomas were excluded. As shown in Table 4, there were three loci showing LRS > 10, one each on chromosome 1, 5 and 16. T-lymphomas in RI strains with F344 allele at the loci on chromosome 5 and 16 had a significantly shorter latency. In contrast, T-lymphomas in RI strains with F344 allele at the locus on chromosome 1 had a slightly longer latency period. The permutation test revealed that this locus fell within the suggestive threshold values linked with the length of the latency period.

### Analysis with backcross rats

Administration of PNU to 139 backcross rats to LE/Stm, 137 (98.6%) developed lympho-haemopoietic tumours, of which 94 were T-lymphomas and 43 erythroleukaemias. As the number of independent LEXF RI strains was limited, we analysed genotype of the backcross rats bearing each type tumours at the loci with any suggestion of linkage for T-lymphoma incidence and their latent period from the RI study. As summarized in Table 5, significant linkage was observed at loci on chromosome 5 ($\chi^2$ = 23.6 at *D5Mit7*), chromosome 7 ($\chi^2$ = 17.7 at *D7Mit4*) and chromosome 10 ($\chi^2$ = 14.5 at *D10Mgh6*) respectively. Weak linkage was found at *D1Mit13* ($\chi^2$ = 7.1). Among the backcross rats, T-lymphomas were more frequent in heterozygotes for the loci on chromosome 5, 7 and 1, but less in LE allele homozygotes for the locus on chromosome 10. This was consistent with the observation in LEXF RI strains (Table 2). The loci on chromosomes 1 and 5 were identified in RI strains as loci affecting length of latency; however, in the backcross, there was no significant difference in latency by genotype at these loci (data not shown). To find other host genes involved in lymphoma type determination, a genome wide screening of the backcross population was carried out, but no linkage with $\chi^2$ > 7 was found except for three loci suggested by analysis of RI strains.

### DISCUSSION

The type of lymphomas in rodents is determined either by lymphomagenic agents or by host genetic constitution. In mice, the primary determinant for T-lymphomas in AKR is the mink cell focus forming (MCF) virus (Cloyd et al, 1980), which acquires thymotropism and leukaemogenicity by successive recombinations among endogenous proviruses (Stoye et al, 1991). Provirus integration adjacent to certain host genes is also a determinant of lymphoma/leukaemia types (Corcoran et al, 1984; Van Lohuizen and Berns, 1990; Copeland and Jenkins, 1999). On the other hand, effects of host genes on lymphoma type determination have been amply recognized. Studying spontaneous lymphomas in AKXD RI strains, Gilbert et al (1993) found that presence of MCF virus and susceptibility allele of *Rmcf* (resistance to MCF virus) are important for high T-lymphoma incidence. Also several other loci linked to *Car-2* (chromosome 3) and *Pmv-25* (chromosome 4) associate with high T-lymphoma frequency and *Mtv-6* (chromosome 16), with lower frequency (Gilbert et al, 1993). The inbred strain SL/Kh is highly predisposed to pre-B-lymphomas (Shimada et al, 1993). In the cross between AKR and SL/Kh (Yamada et al, 1994*b*), we mapped a dominant gene *Tism1* on mouse chromosome 7, which favours T-lymphoma over B-lymphoma susceptibility. An AKR allele of *Tlsm1* seems involved also in a

© Cancer Research Campaign 1999 

predominance of T-lymphomas in some AKXD RI strains (Yamada et al, 1994*b*). In the mice injected subcutaneously with methylcolanthrene, the allelotype of aryl hydrocarbon hydroxylase locus determines whether the tumours are lymphomas or sarcomas at the injection site (Duran-Reynals, 1978). Eμ-*myc* transgene induce B-lineage lymphomas (Harris et al, 1988) in many mouse strains but in C3H, T-lymphomas occur predominantly (Yukawa et al, 1989), although the host gene responsible has not been identified. Similar genetic determination of T-lymphomas is also indicated in rats (Shisa and Hiai, 1985). Unlike mouse models, involvement of endogenous retrovirus is less likely in the rat. The action of these host genes is variable: e.g. affecting metabolic activation of carcinogen, virus propagation, DNA repair, differentiation or proliferation of target cells, cell interactions in thymic microenvironments, or immunologic surveillance to virus or tumours. The genetic determination of disease types seems much more intriguing than initially assumed.

Ogiu et al (1982) and we (Shisa and Hiai, 1985) found that F344 rats have high susceptibility to T-lymphoma induction by PNU, while erythroid or myeloid leukaemias are predominant in most other rat strains. Based on F2 and backcross data between F344 and LE/Stm, we initially assumed that a single dominant gene of F344 determines susceptibility to T-lymphomas (Shisa and Hiai, 1985). In LEXF RI strains, however, the incidence of T-lymphomas showed a continuous array, suggesting involvement of multiple genes in the type determination. To screen genes associated with high T-lymphoma incidence and length of latency period, we performed QTL analysis. In QTL analysis, greater precision is obtained when larger sample size (number of RI strains in this case) is employed. To satisfy the criteria for statistical significance, a marker locus should account for 40% of the genetic variance in a QTL analysis with ten strains, but for only 20% in the same analysis with 20 strains (Plomin et al 1991). In this study, we used a set of 23 substrains, of which 11 were independent. A relatively small number of independent RI strains poses a limitation on obtaining a high level of LRS, therefore, the QTL analysis performed in LEXF RI strains were regarded as a preliminary approach. To minimize type I error, all of potential QTLs were re-evaluated by analysing data in the (F344 × LE) × LE backcross generation.

From the linkage analysis using Map Manager QT software, seven loci were suggested by using the incidence and the length of the latency of T-lymphoma development as parameters. Three of them were found significantly associated with T-lymphomagenesis in 137 backcross rats. The first is a locus linked to *D5Mit7*, which was originally identified by its effect on latency period. In backcross analysis, the F344 allele at *D5Mit7* highly significantly favoured for T-lymphomas ($P = 1 \times 10^{-6}$), but the effect on lymphoma latency was not significant. We postulated that it was due to close correlation between T-lymphomas incidence and the length of latency. In this segment, oncogene *jun* is located in the order of *D5Mit4-D5Mit5-jun-D5Mit7* (Pravenec et al, 1996). *Jun* oncogene is essential to the efficient growth of certain tumours (Angel et al, 1988). The second was *D7Mit4*, closely linked to cellular oncogene *myc*. The gene order is *D7Mit4*-(2.9 cM)-*myc*-(10 cM)-*D7Mit5* (Pravenec et al, 1996). Similarly, F344 allele at this locus favoured to form T-lymphoma ($P = 0.00002$). C-*myc* plays a key role in the control of normal proliferation, transformation and differentiation. It is activated either by translocation (Janz et al, 1993) or by retroviral integration (Selten et al, 1984) and is thus involved in murine lymphomagenesis. The third gene is linked to *D10Mgh6*, the LE allele homozygotes of this locus had a higher T-lymphoma incidence ($P = 0.0001$).

Previously we reported that PNU-induced T-lymphomas in rats is determined by a dominant gene *Tls1* weakly linked with *albino* (*c*) locus (Shisa and Hiai, 1985). In spontaneous lymphoma models in mice, we found that the major dominant gene *Tlsm1* determining development of T-lymphomas is located on mouse chromosome 7, containing the syntenic segment of rat chromosome 1 (Yamada et al, 1994*b*). Therefore, we paid special attention to rat chromosome 1. In the LEXF system, presence of a QTL affecting the length of latency was suggested to associate with *D1Mit5*, but this locus had a small effect on the T-lymphoma development. Investigation of this segment of chromosome 1 in backcross rats revealed a suggestive linkage with *D1Mit13* ($\chi^2 = 7.1$), located at 17.3 cM distal from *c* locus.

The loci on chromosome 4, 16 and 18 identified in RI strains did not show linkage by analysis of backcross rats. Effect of *MHC* loci on lymphoma latency is controversial in mouse models (Lilly and Pincus, 1973; Chen and Lilly, 1982; Mucenski et al, 1986; Vasmel et al, 1988; Kamoto et al, 1996). Resistance to virus or tumour cells by the immune system is regulated by class II *MHC* loci. In LEXF RI strains and backcross rats, however, no significant effect of RT locus was observed (data not shown).

The RI strains of mice have been much used in the genetic study of cancer, including applications to hemopoietic tumours of spontaneous origin (Dux et al, 1978; Bedigian et al, 1981; Gilbert et al, 1993; Yamada et al 1994*b*) or those induced by virus (Silver and Fredrichson, 1983), radiation (Okumoto et al, 1990) and chemicals (Potter et al, 1975). However, to our knowledge, this is the first extensive study of chemical-induced lymphomagenesis with rat RI strains. PNU was given to newly established LEXF RI strains rats. All of the LEXF rats were highly susceptible to hemopoietic malignancies but their types varied remarkably by substrain. Preliminary QTL analysis identified several host genes affecting susceptibility to T-lymphomas and length of latency period. Those loci were further confirmed by analysis of backcross rats derived from the same parental strains. The number of independent RI substrains is small, the precision of gene mapping is limited but the information of such loci is still valuable for further investigation. A variety of non-haemopoietic tumours developed in RI rats, of which duodenal cancers were representative. These tumours developed concomittantly or slightly later than haemopoietic tumours, so that genetic analysis for them is difficult. The most promising use of the LEXF RI strains would be to dissect host genes involved in haemopoietic tumours.

### ACKNOWLEDGEMENTS

This work is supported by Grants-in-Aid for basic study and cancer research from the Ministry of Education, Culture, Sports and Science, Japan, a grant for cancer research from the Ministry of Health and Welfare, Japan, and a grant from the Japan Owner's Association.

### REFERENCES

Angel P, Allegretto EA, Okino ST, Hattori K, Boyle WJ, Hunter T and Karin M (1988) Oncogene *jun* encodes a sequence-specific trans-activator similar to AP-1. *Nature* **322**: 166–170

Bailey DW (1981) Recombinant inbred strains and bilinear congenic strains. In *The Mouse in Biomedical Research*: *History, Genetics and Wild Mice*, Foster HL, Small JD and Fox JG (eds), pp. 223–239. Academic Press: New York

© Cancer Research Campaign 1999

Bedigian HG, Taylor BA and Meier H (1981) Expression of murine leukemia virus in the high lymphomatous BXH-2 recombinant inbred mouse strain. *J Virol* **39**: 632–640

Belknap JK (1992) Empirical estimates of Bonferroni corrections for use in chromosome mapping studies with the BXD recombinant inbred strains. *Behav Genet* **22**: 677–684

Belknap JK, Mitchell SR, O'Toole LA, Helms ML and Crabbe JC (1996) Type I and type II error rates for quantitative trait loci (QTL) mapping studies using recombinant inbred mouse strains. *Behav Genet* **26**: 149–160

Chen S and Lilly F (1982) Suppression of spontaneous lymphoma by previously undiscovered dominant genes in crosses of high- and low-incidence mouse strains. *Virology* **118**: 76–85

Cloyd MW, Hartley JW and Rowe WP (1980) Lymphomagenicity of recombinant mink cell focus-inducing murine leukemia viruses. *J Exp Med* **151**: 542–552

Copeland NG and Jenkins NA (1999) Myeloid leukemia: disease genes and mouse models. *Prog Exp Tumor Res* (in press)

Corcoran LM, Adams JM, Dunn AR and Cory S (1984) Murine T lymphomas in which cellular *myc* oncogene has been activated by retroviral insertion. *Cell* **37**: 113–122

Duran-Reynals ML, Lilly F, Bosch A and Blank KJ (1978) The genetic basis of susceptibility to leukemia induction in mice by 3-methylcholanthrene applied percutaneously. *J Exp Med* **147**: 459–469

Dux A, Muhlbock O and Bailey DW (1978) Genetic analyses of differences in incidence of mammary tumors and reticulum cell neoplasms with the use of recombinant inbred lines of mice. *J Natl Cancer Inst* **61**: 1125–1129

Gilbert DJ, Neumann PE, Taylor BA, Jenkins NA and Copeland NG (1993) Susceptibility of AKXD recombinant inbred mouse strains to lymphomas. *J Virol* **67**: 2083–2090

Harris AW, Pinkert CA, Crawford M, Langdon WY, Brinster RL and Adams JM (1988) The Eμ-*myc* transgenic mose. A model for high incidence of spontaneous lymphoma and leukemia of early B cells. *J Exp Med* **167**: 353–371

Janz S, Muller J, Shaughnessy J and Potter M (1993) Detection of recombinations between c-*myc* and immunoglobulin switch alpha in murine plasma cell tumors and preneoplastic lesions by polymerase chain reaction. *Proc Natl Acad Sci USA* **90**: 7361–7365

Kamoto T, Shisa H, Abujiang P, Lu LM, Yoshida O, Yamada Y and Hiai H (1996) A quantitative trait locus in major histocompatibility complex determining latent period of mouse lymphomas. *Jpn J Cancer Res* **87**: 401–404

Lander E and Kruglyak L (1995) Genetic dissection of complex traits: guidelines for interpreting and reporting linkage results. *Nat Genet* **11**: 241–247

Lilly F and Pincus T (1973) Genetic control of murine viral leukemogenesis. *Adv Cancer Res* **17**: 231–277

Lu LM, Ogawa M, Kamoto T, Yamada Y, Abujiang P and Hiai H (1997) Expression of LECAM-1 and LFA-1 on pre-B lymphoma cells but not on preneoplastic pre-B cells in SL/Kh mice. *Leuk Res* **21**: 337–342

Lu LM, Shisa H and Hiai H (1998) Further characterization of LEXF RI strains of rat. *Rat Genome* **4**: 13–25

Manly KF (1993) A Macintosh program for storage and analysis of experimental genetic mapping data. *Mammal Genome* **4**: 303–313

Manly KF (1996) *Map Manager QT Manual (Version 4)*, pp. 1–20, obtained electronically at net site http://mcbio.med.buffalo.edu/mapmgr.html

Mucenski M/L, Taylor BA, Jenkins NA and Copeland NG (1986) AKXD recombinant inbred strains: models for studying the molecular genetic basis of murine lymphomas. *Mol Cell Biol* **6**: 4236–4243

Ogiu T and Odashima S (1982) Induction of rat leukemias and thymic lymphomas by N-nitrosoureas. *Acta Pathol Jap* **32**: 223–235

Okumoto M, Nishikawa R, Imai S and Hilgers J (1990) Genetic analysis of resistance to radiation lymphomagenesis with recombinant inbred strains of mice. *Cancer Res* **50**: 3848–3850

Plomin R, McClearn GE, Gora-Maslak G and Neiderhiser JM (1991) Use of recombinant inbred strains to detect quantitative trait loci associated with behavior. *Behav Genet* **21**: 99–116

Potter M, Pumphrey JG and Bailey DW (1975) Genetics of susceptibility to plasmacytoma induction. I. BALB/cAnN (C), C57BL/6N (B6), C57BL/Ka (BK), (C times B6)F1, (C times BK)F1 and C times B recombinant-inbred strains. *J Natl Cancer Inst* **54**: 1413–1417

Pravenec M, Gauguier D, Schott JJ, Buard J, Kren V, Bila V, Szpirer C, Wang JM, Huang H, Lezin ES, Spence MA, Flodman P, Printz M, Lathrop GM, Vergnaud G and Kurtz TW (1996) A genetic linkage map of the rat derived from recombinant inbred strains. *Mammal Genome* **7**: 117–127

Selten G, Cuypers HT, Zijlstra M, Melief C and Berns A (1984) Involvement of c-myc in MuLV-induced T cell lymphomas in mice: frequency and mechanisms of activation. *EMBO J* **3**: 3215–3222

Shimada MO, Yamada Y, Nakakuki Y, Okamoto K, Fukumoto M, Honjo T and Hiai H (1993) SL/Kh strain of mice: a model of spontaneous pre-B lymphomas. *Leuk Res* **17**: 573–578

Shisa H and Hiai H (1985) Genetically determined susceptibility of Fischer 344 rats to propylnitrosourea-induced thymic lymphomas. *Cancer Res* **45**: 1483–1487

Shisa H and Suzuki M (1991) Action site of the gene determining susceptibility to propylnitrosourea-induced thymic lymphomas in F344 rats. *Jpn J Cancer Res* **82**: 46–50

Shisa H, Lu LM, Katoh H, Kawarai A, Tanuma J, Matsushima Y and Hiai H (1997) The LEXF: a new set of recombinant inbred strains between LE/Stm and F344 *Mammal Genome* **8**: 324–327

Silver JE and Fredrickson TN (1983) Susceptibility to Friend helper virus leukemias in CXB recombinant inbred mice. *J Exp Med* **158**: 1693–1702

Stoye JP, Moroni C and Coffin JM (1991) Virological events leading to spontaneous AKR thymomas. *J Virol* **65**: 1273

Taylor BA (1978) Recombinant inbred strains: use in gene mapping. In *Origin of Inbred Mice*, Morse HC III (ed), pp. 423–438. Academic Press: New York

Van Lohuizen M and Berns A (1990) Tumorigenesis by slow-transforming retrovirus: an update. *Biochim Biophys Acta* **1032**: 213–235

Vasmel WL, Zijlstra M, Radaskiewicz T, Leupers CJ, De Goeda RE and Melief CJ (1988) Major histocompatibility complex class II regulated immunity to murine leukemia virus protects against early T-but not late B-cell lymphomas. *J Virol* **62**: 3156–3166

Yamada Y, Matsushiro H, Ogawa MS, Okamoto K, Nakakuki Y, Toyokuni S, Fukumoto M and Hiai H (1994*a*) Genetic predisposition to Pre-B lymphomas in SL/Kh strain mice. *Cancer Res* **54**: 403–407

Yamada Y, Shisa H, Matsushiro H, Kamoto T, Kobayashi Y, Kawarai A and Hiai H (1994*b*) T lymphomagenesis is determined by a dominant host gene *Thymic Lymphoma Susceptible Mouse-1 (TLSM-1)* in mouse model. *J Exp Med* **180**: 2155–2162

Yukawa K, Kikutani H, Inomoto T, Uehira M, Bin SH, Akagi K, Yamamura K and Kishimoto T (1989) Strain dependency of B and T lymphoma development in immunoglobulin heavy chain enhancer *Eμ-myc* transgenic mice. *J Exp Med* **170**: 711–726

*© Cancer Research Campaign 1999*
